# Kanglaite attenuates UVB-induced down-regulation of aquaporin-3 in cultured human skin keratinocytes

**DOI:** 10.3892/ijmm.2011.873

**Published:** 2011-12-29

**Authors:** SHI-JUN SHAN, TING XIAO, JOHN CHEN, SHI-LING GENG, CHANG-PING LI, XUEGANG XU, YUXIAO HONG, CHAO JI, YING GUO, HUACHEN WEI, WEI LIU, DAPENG LI, HONG-DUO CHEN

**Affiliations:** 1 Department of Dermatology, Tianjin Medical University General Hospital, Tianjin; 2 Department of Dermatology, The No. 1 Hospital of China Medical University, Shenyang, P.R. China; 3 Dermatology Associates, Tucson, AZ, USA; 4 Department of Dermatology, Heihe People Hospital, Heihe, Heilongjiang; 5 Department of Health Statistics, College of Public Health, Tianjin Medical University, Tianjin; 6 Department of Dermatology, The First Affiliated Hospital of Nanjing Medical University, Nanjing, P.R. China; 7 Ackerman Academy of Dermatopathology, New York, NY; 8 Dermatology Research Laboratories, Department of Dermatology, The Mount Sinai Medical Center, New York, NY, USA; 9 Department of Dermatology, Air Force General Hospital, Beijing; 10 Zhejiang Kanglaite Pharmaceutical Co., Ltd., Hangzhou, Zhejiang, P.R. China

**Keywords:** Kanglaite, photoaging of skin, aquaporin-3

## Abstract

Ultraviolet (UV) radiation plays an important role in the pathogenesis of skin photoaging. Depending on the wavelength of UV, the epidermis is affected primarily by UVB. One major characteristic of photoaging is the dehydration of the skin. Membrane-inserted water channels (aquaporins) are involved in this process. In this study we demonstrated that UVB radiation induced aquaporin-3 (AQP3) down-regulation in cultured human skin keratinocytes. Kanglaite is a mixture consisting of extractions of Coix Seed, which is an effective anti-neoplastic agent and can inhibit the activities of protein kinase C and NF-κB. We demonstrated that Kanglaite inhibited UVB-induced AQP3 down-regulation of cultured human skin keratinocytes. Our findings provide a potential new agent for anti-photoaging. The related molecular mechanisms remain to be further elucidated.

## Introduction

Skin aging is a degenerative process including intrinsic aging which is characterized by dryness, generalized wrinkling and thin appearance. Solar ultraviolet (UV) radiation is the most significant extrinsic factor which causes photoaging of the skin, manifesting as deep wrinkling, severe roughness and dryness. The photoaging of the skin partly overlaps and superimposes the intrinsic dryness (1). Solar UV reaching earth is comprised of UVA (320–400 nm in wavelength) and UVB (280–320 nm). UVB is mostly absorbed by the epidermis and predominantly affects keratinocytes (1,2).

Water movement across the plasma membrane occurs via two pathways: diffusion through the lipid bilayer and via aquaporins (AQPs) (1,3–5). The AQPs act primarily as water-selective pores and facilitate water transport across cell plasma membranes (6). There are at least 13 mammalian AQPs (AQP0-AQP12), which have been divided into two groups on the basis of their permeability. AQP 1, 2, 4, 5 and 8 are primarily water-selective transporters; AQP 3, 7, 9 and 10 transport water, glycerol and other small solutes (7,8).

It has been demonstrated that AQP3 expressed in the basal layer of the epidermis and the deficiency reduce the stratum corneum hydration and glycerol content (4,8,9). After exposure to UV radiation, AQP3 down-regulation reduces the stratum corneum hydration; the deficient water conditions damage the function of the skin, leading to dryness and wrinkle formation (1,10). However, the functions of AQP3 in human skin keratinocytes remain to be further elucidated.

Adequate photoprotection is essential to prevent UV-related damage. Photoprotective agents, such as polyphenols and baicalin, have been demonstrated to be effective in photoprotection via influencing pertinent cell signaling pathways (11). Kanglaite is a mixture consisting of extractions from Coix seed, a Chinese herb, which has been demonstrated to be effective in anticancer treatment via inhibition of COX-2, MMP9, protein kinase C and NF-κB (12,13). We carried out this study to investigate whether Kanglaite has any protective effects against UVB-induced AQP3 down-regulation in cultured human skin keratinocytes.

## Materials and methods

### UV light apparatus

The UV radiation apparatus used in the study was the Waldmann UV801KL (Waldmann GmbH Co., Germany). The UVB wavelength was 285–350 nm (peak 312 nm). As previously described, (5,15) before UVB radiation, cultured human skin keratinocytes were washed with 1 ml PBS buffer and then changed to 0.5 ml PBS in each well. The cells were radiated at the desired intensity without a plastic dish lid. After UVB radiation, the cells were returned to incubation in basal medium with treatments for various times prior to harvest.

### Chemicals and reagents

Rabbit anti-AQP3 was obtained from Chemicon (Temecula, CA). Monoclonal mouse anti-β-actin was obtained from Sigma (St. Louis, MO). Kanglaite was obtained from the Zhejiang Kanglaite Pharmaceutical Co. (Hangzhou, China).

### Cell culture

As previously described (5,14), spontaneously immortalized human keratinocytes (HaCaT) were maintained in DMEM medium (Sigma) supplemented with 10% fetal bovine serum (Invitrogen, Carlsbad, CA), penicillin/streptomycin (1:10; Sigma) and 4 mM L-glutamine (Sigma), in a CO_2_ incubator at 37°C. For Western blotting, cells were reseeded in 6-well plates at a density of 0.5×10^6^ cells/ml with fresh complete culture medium.

### MTT assay

The cell proliferation effect of Kanglaite was determined by the MTT assay. The cells (4×10^3^ cells/ml) were cultured on a 96-well plate in a DMEM medium with different concentrations of Kanglaite (0.5×10^−4^, 1×10^−3^, 5×10^−3^, 1×10^−2^, 5×10^−2^, 1×10^−1^ ml/ml) for 24 h. The cells were next washed with PBS and 200 μl of MTT (0.05 mg/ml) was added to each well, followed by incubation for 4 h at 37°C. The supernatant was removed, and 200 μl of dimethylsulfoxide was added to each well to dissolve the formazan product. Wells without cells were used as blank controls. Absorbance was determined at 570 nm, spectrophotometrically, using an ELISA reader (Tecan, Salzburg, Austria). The results are expressed as the percentage of control cells obtained from six experiments conducted under the same culture conditions.

### RNA extraction, reverse transcription and PCR

Total-RNA from cells was extracted using the SV total-RNA Isolation System (Zhongshan, China) following the manufacturer’s instructions. The concentration of total-RNA was determined by measuring the optical density at 260 nm. Total-RNA (1 μg) was converted into first-strand cDNA using the ImProm-II Reverse Transcription system with random primers following the manufacturer’s instructions (Zhongshan). Parallel reactions for each RNA sample were run in the absence of reverse transcriptase to assess any genomic DNA contamination of the RNA.

For the semi-quantitative reverse transcription PCR experiment, the product was amplified using specific primers designed as described before (15,16): AQP3 forward, 5′-GCT GTC ACT CTG GGC ATC CTG-3′ and reverse primers, 5′-GCG TCT GTG CCA GGG TGT AG-3′, amplifying a 131-bp product and the GAPDH forward, 5′-TCC TGT GGC ATC CAC GAA ACT-3′ and reverse primers, 5′-GAA GCA TTT GCG GTG GAC GAT-3′, amplifying a 313-bp product.

**Figure 1:**
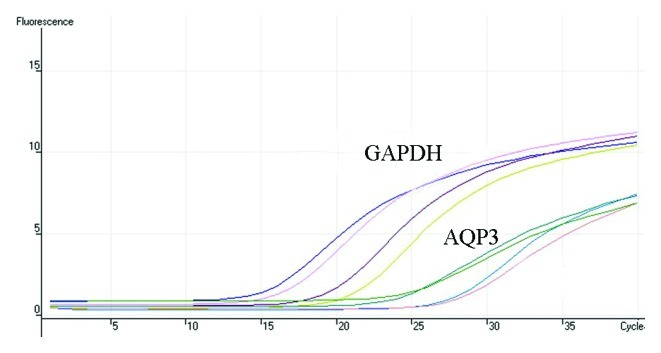
GAPDH and AQP3 real-time quantitative PCR proliferation curves.

The real-time quantitative PCR experiments were carried out in an Rotor-gene 3000 (Australia), using a SYBR-Green PCR Mastermix (Zhongshan). Each sample was analyzed iduplicate along with standard and no-template controls. The reaction contained 30 ng cDNA in 1 μl Mastermix, including pre-set concentrations of deoxyribonucleotide triphosphates, MgCl_2_, and buffers, along with 300 nM forward and reverse primers and the SYBR-Green reporter dye. The PCR parameters were 95°C for 2 min, 40 cycles at 95°C for 15 sec, 60°C for 1 min and 72°C for 30 sec (Fig. 1). RNA concentrations were determined by comparing cDNA-generated signals in samples with those generated from known amounts of cDNA. RNA levels were corrected with the GAPDH cDNA signal for variations in the amounts of input RNA. The product purity was confirmed using a dissociation standard curve.

### Western blot analysis

As reported previously (5,14), cultured skin keratinocytes with or without treatment were washed with cold PBS and harvested by scraping into 100 μl of RIPA buffer. Cell lysates were incubated at 4°C for 30 min. Proteins (20 μg) were denatured in 5X SDS-PAGE sample buffer for 5 min at 95°C. Proteins were separated by 10 or 12% SDS-PAGE gels and transferred onto PVDF membranes (Millipore, Bedford, MA). Nonspecific binding was blocked with 10% dry milk in TBST for 1 h at room temperature. After blocking, membranes were incubated with specific antibodies in dilution buffer (2% BSA in TBS) overnight at 4°C. Blots were incubated with horseradish peroxidase conjugated anti-rabbit or anti-mouse IgG at appropriate dilutions and room temperature for 1 h. Antibody binding was detected using the enhanced chemiluminescence (ECL) detection system (Amersham Biosciences) following the manufacturer’s instructions and visualized by autoradiography with Hyperfilm.

### Statistical analysis

The values in the figures were expressed as the means ± standard error (SE). The data in this study are representative of more than three different experiments. Repeated measures of one factor ANOVA was used to analyze the data. The SNK-q assay was performed between the treated groups. The Student’s t-test was performed to detect differences between the Kanglaite and vehicle groups. P<0.05 was considered significant.

## Results

### Effect of Kanglaite on proliferation in cultured human skin keratinocytes

Cultured skin keratinocytes were treated with 0, 5×10^−4^, 1×10^−3^, 5×10^−3^, 1×10^−2^, 5×10^−2^, 1×10^−1^ ml/ml Kanglaite. The results of the MTT assay showed proliferation rates of 0.093±0.008, 0.963±0.280, 1.140±0.201, 1.073±0.132, 1.055±0.233, 1.068±0.208 and 0.857±0.218, respectively. Kanglaite in all the examined concentrations exhibited no inhibitory effect on the proliferation of cultured skin keratinocytes (P>0.05).

### UVB radiation down-regulates AQP3 mRNA in cultured human skin keratinocytes

**Figure 2:**
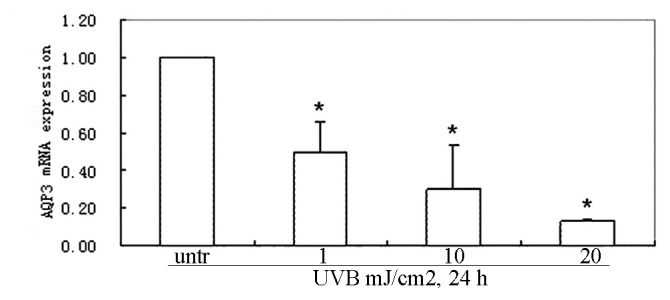

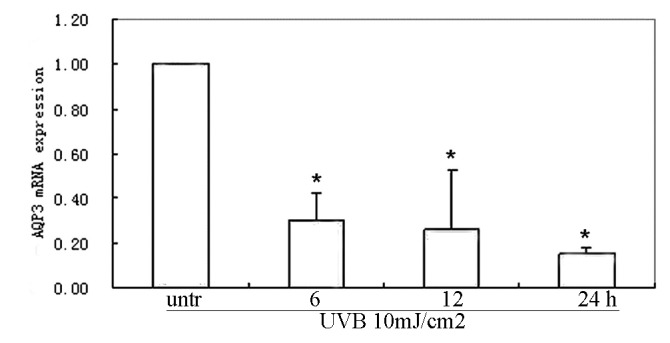
UVB induces down-regulation of AQP3 mRNA expression in a time- and dose-dependent manner in cultured skin keratinocytes (HaCaT cells). (A) HaCaT cells were treated with different UVB doses (1, 10 or 20 mJ/cm^2^) after 24 h of incubation. (B) HaCaT cells were treated with UVB (10 mJ/cm^2^) and the AQP3 gene expression was analyzed by real-time quantitative PCR at different times (6, 12 and 24 h) (B). The differences between the treated groups vs. the untreted group were analyzed by the SNK-q assay. The data represent the mean ± SE of three independent experiments. ^*^P<0.05 vs. the untreated (untr) group.

Cultured skin keratinocytes were radiated with 1, 10, 20 mJ/cm^2^ UVB. Cells were collected after 24 h of culture. The effect of UVB radiation on gene expression was determined by means of real-time quantitative PCR. The results of a relative quantification analysis revealed that the UVB-induced AQP3 mRNA down-regulation was dose-dependent. The AQP3 mRNA expression was down-regulated after radiation with 1 mJ/cm^2^ UVB and the down-regulation was most obvious after 20 mJ/cm^2^ of UVB irradiation (F=19.88, P<0.0005) (Fig. 2A). The UVB-induced down-regulation of AQP3 mRNA was also time-dependent. The AQP3 mRNA expression was first found to be down-regulated at 6 h and was most obvious at 24 h after radiation with 10 mJ/cm^2^ UVB (F=25.30, P<0.0002) (Fig. 2B).

### Kanglaite inhibits UVB-induced down-regulation of AQP3 mRNA in cultured human skin keratinocytes

**Figure 3:**
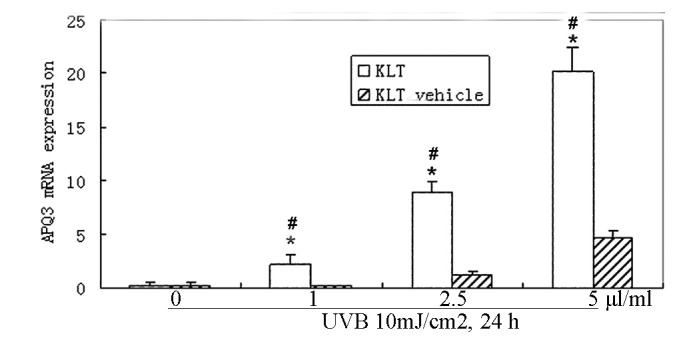

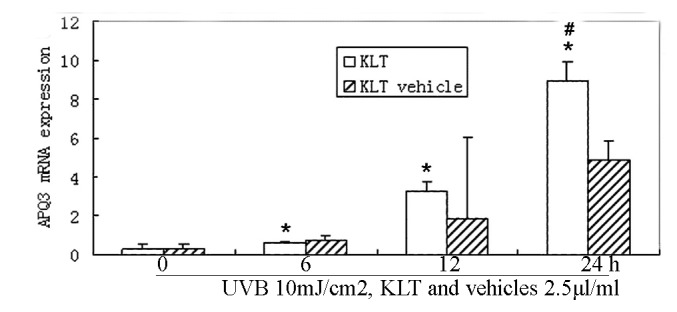
Kanglaite up-regulates the AQP3 gene in a dose-dependent manner in HaCaT cells. (A) HaCaT cells were treated with different doses of Kanglaite (KLT) (1, 2.5 or 5 μl/ml) and the same concentrations of Kanglaite vehicle for 24 h. AQP3 expression was analyzed by real-time quantitative PCR. The differences between the Kanglaite or vehicle treated groups vs. the UV-radiated groups were assessed by the SNK-q assay. The Student’s t-test was used to assess differences between the Kanglaite and vehicle groups. The data represent the mean ± SE of three independent experiments. ^*^P<0.05 vs. the UV-radiated group. ^#^P<0.05 vs. the Kanglaite vehicle group. (B) Kanglaite up-regulates the AQP3 gene in a time-dependent manner in HaCaT cells. HaCaT cells were treated with 2.5 μl/ml Kanglaite and the same concentrations of Kanglaite vehicle for 6, 12 and 24 h. AQP3 gene expression was analyzed by real-time quantitative PCR. The data represent mean ± SE of three independent experiments. ^*^P<0.05 vs. UV-radiated group. ^#^P<0.05 vs. the Kanglaite vehicle group.

Cultured skin keratinocytes were radiated with 10 mJ/cm^2^ UVB. Cells were collected after 24 h of incubation with 1, 2.5 or 5 μl/ml Kanglaite and the same concentrations of Kanglaite vehicles were used as controls. Real-time quantitative PCR was performed. Application of 1 μl/ml Kanglaite significantly up-regulated the AQP3 mRNA expression after 24 h incubation (F=−3.84 P=0.0184); 2.5, 5 μl/ml Kanglaite incubation significantly up-regulated the AQP3 transcripts by 8.92±1.04 and 20.20±2.25-fold, respectively. Significant up-regulation was observed in all 1, 2.5 and 5 μl/ml Kanglaite-treated groups vs. the Kanglaite vehicle groups (Fig. 3A). After 6, 12 or 24 h of incubation with 2.5 μl/ml Kanglaite and the vehicles, significant up-regulation of the AQP3 transcripts was only observed in the 24 h samples (F=−4.80, P=0.0086) (Fig. 3B). There were no significant changes in the 6 and 12 h samples between the Kanglaite and vehicle groups.

### UVB irradiation down-regulates AQP3 protein in cultured skin keratinocytes

**Figure 4:**
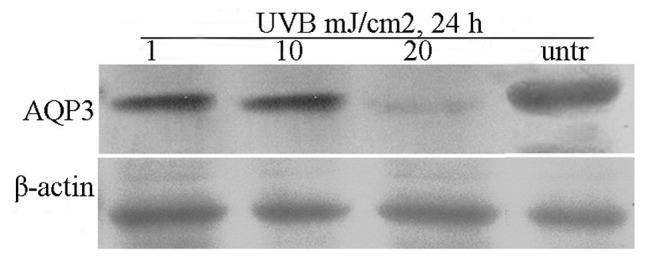

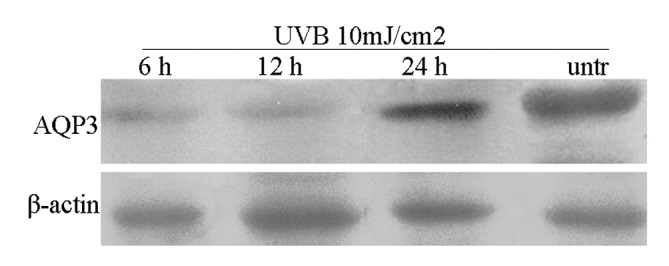
UVB induces AQP3 down-regulation in a time- and dose-dependent manner in HaCaT cells. (A) Cells were treated with different UVB doses (1, 10 or 20 mJ/cm^2^) and AQP3 expression was analyzed by Western blotting after 24 h. (B) HaCaT cells were treated with UVB (10 mJ/cm^2^) and AQP3 was analyzed by Western blotting at different times (6, 12 or 24 h). All experiments were repeated at least three times and similar results were obtained.

Cultured skin keratinocytes were radiated with 1, 10 or 20 mJ/cm^2^ UVB. Cells were collected after 24 h of culture. Western blot analysis showed that UVB irradiation down-regulated the AQP3 protein expression in a dose-dependent manner. AQP3 protein expression began to decrease after 1 mJ/cm^2^ UVB radiation, and more significantly when radiated with 20 mJ/cm^2^ UVB compared to untreated samples (Fig. 4A). After being radiated with 10 mJ/cm^2^ UVB and cultured for 6, 12 or 24 h, AQP3 protein expression was again measured by Western blotting. The results showed that AQP3 began to decrease at 6 h and further decreased at 12 h. After 24 h culture, AQP3 expression began to return towards basal levels compared to that at 12 h (Fig. 4B).

### Kanglaite inhibits UVB-induced down-regulation of AQP3 protein expression in cultured skin keratinocytes

**Figure 5:**
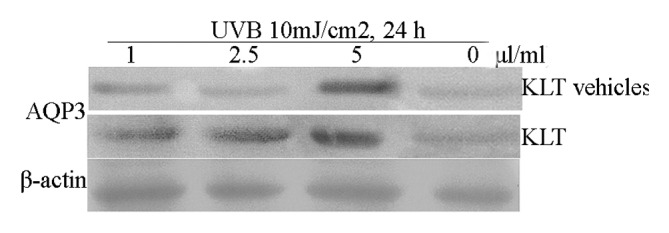

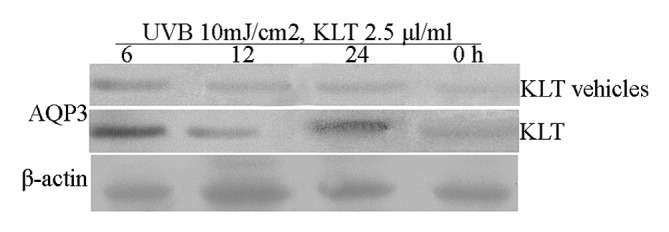
Kanglaite up-regulates AQP3 in a time- and dose-dependent manner in HaCaT cells. (A) HaCaT cells were treated with different doses of Kanglaite (1, 2.5 or 5 μl/ml) and the same concentrations of Kanglaite vehicles for 24 h or (B) treated with Kanglaite (2.5 μl/ml) for different times (6, 12, 24 h). AQP3 expression was analyzed by Western blotting. All experiments were repeated at least three times and similar results were obtained.

To analyze the effect of Kanglaite on AQP3 protein expression after UVB irradiation, cultured skin keratinocytes were radiated with 10 mJ/cm^2^ UVB and incubated with 1, 2.5, 5 μl/ml Kanglaite and Kanglaite vehicles, respectively. Application of 1 μl/ml Kanglaite significantly up-regulated AQP3 protein expression after 24 h incubation; 2.5, 5 μl/ml Kanglaite also significantly up-regulated the AQP3 protein expression (Fig. 5A). After 6, 12 or 24 h of incubation with 2.5 μl/ml Kanglaite and the vehicles, significantly up-regulated AQP3 protein expression was observed in the Kanglaite-treated groups. No significant changes in AQP3 protein expression were observed in the vehicle-treated groups (Fig. 5B).

## Discussion

Some intracellular and extracellular proteins are involved in skin photoaging through regulation of cell signaling pathways such as JNK, ERK, p38 MAP kinase and PI3K/AKT kinases (14,17). Antioxidants and botanical agents such as polyphenols, epigallocathechin-3-gallate, flavonoids, isoflavonoids and all-trans retinoic acid have been demonstrated to be able to block certain pathways to exert their protective effect on skin photoaging (14,18–21).

Dehydration is one of the major events of skin photoaging. Previous studies have shown that AQP3 plays an important role in the regulation of water permeability (22). UV radiation down-regulates AQP3 expression in cultured skin keratinocytes, then decreases water permeability of keratinocytes. Meanwhile the reduction of water permeability is also due to the reduced glycerol transport through AQP3 (14).

UV-induced H_2_O_2_, oxidized lipid hydroperoxides and ROS also induce AQP3 down-regulation via activating the MEK/ERK pathway in cultured skin keratinocytes (23). The MEK/ERK pathway is one of the most important pathways in mediating the UV-induced skin photoaging and skin cancer (5,14,24).

In the present study, the results showed that UVB radiation down-regulated AQP3 mRNA and protein expression in a dose and time-dependent manner in cultured skin keratinocytes, which was in accordance with previous reports (5,14).

trans-Zeatin (tZ), retinoids and nicotinamide can up-regulate skin AQP3 expression (17,19,25). In a previous study it was found that tZ inhibits UV-induced MEK-ERK activation. tZ up-regulates AQP3 expression in a dose and time-dependent manner (5). In another study, UV radiation was shown to down-regulate AQP3 expression in cultured skin keratinocytes via reactive oxygen species mediated MEK/ERK pathways. All-trans retinoic acid inhibits the UV-induced AQP3 down-regulation and increases the water permeability of cultured keratinocytes (14). This may provide us the new agents with protective effects against UV-induced photoaging.

The Coix seed has long been used in traditional Chinese medicine for treatment of various diseases, particularly cancer and skin HPV infection. Kanglaite injection is an acetone extract of herbal medicine Coix seed using high performance liquid chromatography pharmaceutical technology. The injection has been approved for the treatment of lung, hepatic, colon, prostate, and esophageal cancer via inhibiting tumor cell mitosis at the boundary of the G2/M phase and inducing apoptosis through activation of the Fas/FasL pathway. Kanglaite treatment results in a significant down-regulation of PTGS2 mRNA, the gene which encodes COX-2 (12). Kanglaite significantly inhibits the growth of human MDA-MB-231 breast cancer cell via inhibiting NF-κB signaling and protein kinase C activity (13).

In this study, we found that Kanglaite inhibits UVB-induced down-regulation of AQP3 expression. The mode of action of Kanglaite is unlike certain ingredients of some cosmetics products which claim to increase epidermal AQP3 expression, and in fact, high AQP3 level may be associated with high risk of skin tumors (7,26). Our findings may provide a new agent with protective effects against UV-induced photoaging and may contribute to potential therapeutic strategies for the treatment and prevention of skin photoaging. The mechanism of the inhibitory effect of Kanglaite needs to be further investigated.
